# NTN-1 attenuates amyloid-β-mediated microglial neuroinflammation and memory impairment via the NF-κB pathway and NLRP3 inflammasome in a rat model of Alzheimer's disease

**DOI:** 10.3389/fnagi.2025.1516399

**Published:** 2025-04-28

**Authors:** Tianhang Wang, Yanchen Liu, Yidan Lu, Lijun Chi

**Affiliations:** Department of Neurology, The First Affiliated Hospital of Harbin Medical University, Harbin, China

**Keywords:** netrin-1, microglia, neuroinflammation, NLRP3 inflammasome, NF-κB, Alzheimer's disease (AD), apoptosis-associated speck like protein (ASC), amyloid-β (Aβ)

## Abstract

**Introduction:**

Neuroinflammation driven by microglial activation represents a pivotal pathological mechanism underlying brain injury in Alzheimer's disease (AD), with NLRP3 inflammasome activation being a hallmark feature of this process. Netrin-1 (NTN-1) was recently shown to have potent anti-inflammatory and anti-apoptotic properties in a range of inflammatory diseases; however, its potential effect on neuroinflammation in AD treatment has not been well examined. Accordingly, this study aimed to investigate the effects of NTN-1 on cognitive impairment and to explore the anti-inflammatory properties related to the NLRP3 inflammasome and NF-κB signaling in Aβ1-42-induced rat models.

**Methods:**

We assessed the effects of NTN-1 on neurobehavioral function, microglial activation and neuroinflammation mechanisms in Aβ1-42-treated rats using the Morris water maze test and Western blotting.

**Results:**

Our results indicated that microinjections of NTN-1 attenuated Aβ1-42-induced memory and cognitive dysfunction and significantly inhibited microglial proliferation and NLRP3 inflammasome activation in the hippocampus and cortex of AD rats. Additionally, NTN-1 effectively prevented proinflammatory factor (IL1β and IL18) release and NF-κB signaling upstream activation.

**Discussion:**

Overall, the results of the present study indicated that exogenous NTN-1 treatment prevented neuroinflammation and cognitive deficits by inhibiting microglial activation, which is possibly mediated by the NF-κB signaling pathway and NLRP3 inflammasome activation in Aβ1-42-simulated rat models. NTN-1 emerges as a promising therapeutic candidate for mitigating microglia-mediated neuropathology in AD through its anti-inflammatory properties.

## Introduction

The most prevalent form of dementia, Alzheimer's disease (AD), is characterized by amyloid-β (Aβ) accumulating in oligomers and senile plaques in the central nervous system (CNS) (Lane et al., 2018). Chronic deposition of Aβ peptides within the CNS, particularly in microglia, triggers neuroimmune cascades that critically contribute to AD pathogenesis through sustained neuroinflammation (Webers et al., 2020). Activated microglia proliferate and secrete proinflammatory cytokines, including the key cytokines interleukin-1β (IL-1β), interleukin-18 (IL-18) and tumor necrosis factor (TNF)-α, which eventually cause neuroimmune damage, Aβ plaque accumulation in neurons, neurotoxicity, progressive behavioral abnormalities and loss of memory symptoms in AD (Hansen et al., 2018; Nanjundaiah et al., 2021).

The transcription and production of the inactive precursor IL-1β is mediated by the activation of nuclear factor-κB (NF-κB), one of the classical signaling pathways in the microglial immune response (Jiang et al., 2015; Li et al., 2019). Moreover, pro-IL-1β and pro-IL-18 are processed into biologically active forms to play a role in neuroinflammation by protease caspase-1, a member of the NLRP3 inflammasome (Halle et al., 2008; Kelley et al., 2019). Recently, the NLRP3 inflammasome has been established to be a novel inflammasome signaling pathway in response to various stimuli such as infection, cell damage or Aβ fibrils (Thawkar and Kaur, 2019). The inflammasome comprises the cytoplasmic receptor NLRP3, apoptosis-associated speck-like protein (ASC) and caspase-1 downstream (Spel and Martinon, 2020). Activation of the NLRP3 inflammasome requires a two-step process: NF-κB-mediated NLRP3 transcription and subunit assembly of the NLRP3 inflammasome by upstream activating molecules (Bauernfeind et al., 2009; Dun and Parkinson, 2017). It has been demonstrated that cleaved caspase-1 expression is strongly higher in human AD brains, and NLRP3 or caspase-1 gene knockout AD mice have less memory impairment and enhanced Aβclearance (Heneka et al., 2013). NLRP3 is also reported to be active and produce IL-1β along with microglial activating and neurotoxic factors aggregating in Aβ-driven models (Heneka et al., 2013; Tschopp and Schroder, 2010). These results suggest that the NF-κB-NLRP3 inflammasome pathway mediates neuroimmune damage and amyloid-beta production and is proposed as an alternative strategy in the treatment of AD.

Netrin-1 (NTN-1), an extracellular protein that has been shown to regulate cell migration, guide axonal growth and exert anti-inflammatory functions in the CNS (Lou et al., 2020; Mulero et al., 2017). Several studies have observed that altering the expression of NTN-1 has beneficial effects in tumors, autoimmune disorders, ischemic stroke and Parkinson's disease (El-Gamal et al., 2020; He et al., 2018; Moon et al., 2006; Tadagavadi et al., 2010). Spilman PR et al. found that overexpression of NTN-1 in transgenic AD mice reduced both Aβ1-42 and Aβ1-40 and improved cognition (Spilman et al., 2016). Intrahippocampal fissure-injected NTN-1 prevented memory impairment in an Aβ1-42-related rat model (Zamani et al., 2019). In our previous study, we also found that the expression of NTN-1 was decreased in both the cerebrospinal fluid (CSF) and serum of Aβ-induced AD rats (Sun et al., 2019). It was demonstrated that the expression of NTN-1 is associated with amyloid-beta production, which might be a key factor in Aβ regulation (Lourenço et al., 2009). NTN-1 strongly exerts an anti-inflammatory properties by suppressing NF-κB pathway activation in endothelial cells and subarachnoid hemorrhage rats, and it also plays a critical role in preventing NF-κB and caspase-3/7 activation and ROS damage by microinjection in AD rats and SH-SY5Y cells (Lin et al., 2018; Xie et al., 2018; Zamani et al., 2020, 2019). Moreover, it was reported that NTN-1 reduced IL-1β and IL-12β and increased PPARγ expression in cultured astrocytes after brain injury (He et al., 2018). Despite this evidence, the detailed molecular mechanism of NTN-1 in Aβ1-42-simulated neuroinflammation regulation and neuroprotection in AD was previously unknown. Herein, we focused on exploring the molecular mechanisms of NTN-1 in the anti-inflammatory and neuroprotective effects of Aβ1-42-induced AD rats.

## Materials and methods

### Animals

Adult male Sprague–Dawley rats (300~350 g) from the center of experimental animals at Harbin Medical University of China were housed under standard laboratory conditions (T: 25 ± 1°C, a 12-h light/dark cycle). Before the experiment, rats were kept for 7 days to adapt to their environment and had free access to food and water available *ad libitum*. All animal experimental procedures were performed strictly in accordance with the Animal Ethics Committee of Harbin Medical University.

### Aβ1-42 and NTN-1 treatment preparation

The rat Aβ1-42 peptide (SCP0038, Sigma) was dissolved in sterilized saline to achieve the desired concentration (5.5 μg/μL). It was equipped in several microtubes and stored at −20°C. Before use, the peptide was incubated for 1 week at 37°C (Zhang et al., 2013). NTN-1 (R&D) powder was dissolved in sterilized saline, vortexed to a final concentration of 160 ng/μL and stored at −20°C before use (Zamani et al., 2019).

### Rat microinjection and surgery

**Figure 1:**
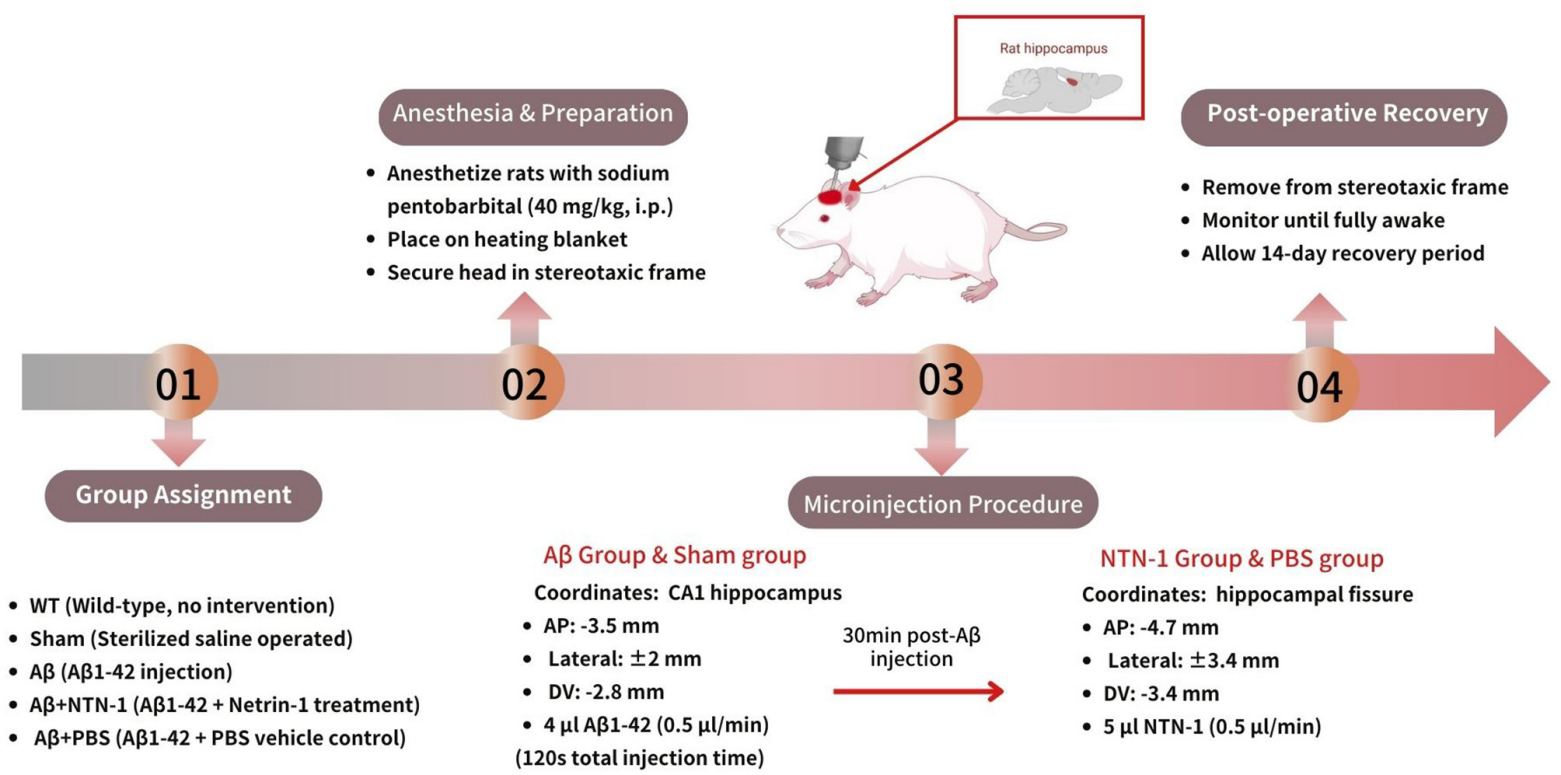
Hippocampal microinjection of Aβ1–42 and Netrin-1 intervention in rats. Adult male rats were stratified into five groups (15/group): wild-type (WT), sham (sterile saline-injected), Aβ1–42 (Aβ), Aβ1–42 + Netrin-1 (Aβ+NTN-1), and Aβ1–42 + PBS vehicle control (Aβ+PBS). Under sodium pentobarbital anesthesia (40 mg/kg, i.p.), Aβ1–42 (4 μL) or equivalent sterile saline/PBS was stereotaxically infused into bilateral hippocampal CA1 regions (coordinates: −3.5 mm AP, ±2.0 mm ML, −2.8 mm DV) at 0.5 μL/min. For the Aβ+NTN-1 group, 5 μL Netrin-1 was administered into hippocampal fissures (AP: −4.7 mm, ML: ±3.4 mm, DV: −3.4 mm) 30 min post-Aβ injection. Sham and vehicle groups received matched volumes of saline or PBS at identical coordinates. Postoperative recovery spanned 14 days.

The animals were stratified into the following groups: wild-type group(WT), sterilized saline operated group (sham), Aβ 1-42 group (Aβ), Netrin-1 treatment Aβ 1-42 group (Aβ+NTN-1) and vehicle control Aβ1-42 group (Aβ+PBS), with similar numbers of male rats included (15 rats per group). Age-matched adult male animals of similar weights were anesthetized with sodium pentobarbital (40 mg/kg) by intraperitoneal injection. Rats were placed on a heating blanket to keep the body temperature, the head was fixed in a stereotaxic frame, and microinjection was performed as previously described (Zamani et al., 2019). Briefly, 4 μL Aβ1-42 sterilized saline were slowly injected (0.5 μL/min) into CA1 region of each hippocampus using a Hamilton syringe at a position: −3.5 mm lateral to bregma, ±2 mm from midline, and −2.8 mm deep from the surface of the skull over a period of 120 s. For NTN-1 group, 5 μL NTN-1 buffer was slowly injected into each hippocampal fissure (0.5 μL/min) using the same syringe 30 min later at the following position (AP: −4.7 mm, laterality: ±3.4 mm, and DV: −3.4 mm). For sham and vehicle control groups, equal volumes of sterile saline or PBS were injected into the respective areas instead according to the coordinates that mentioned earlier, followed by a 14-day rest to allow for recovery (Figure 1).

### Morris water maze task

Spatial learning and memory were assessed on Day 16 postsurgery by the Morris water maze (MWM) task as previously described (Ai et al., 2013). Briefly, a 2.0 m diameter black circular pool was filled with plain water (24 ± 1°C) and divided into four quadrants. A transparent platform (20 cm diameter) was submerged 2 cm under the water surface and placed in the first quadrant. Prior to testing, the rats received three learning trials per day for 5 consecutive days to adapt to the hidden platform. For each trial, the rats started from a random quadrant (not the target quadrant) to swim freely for 60 s and then rested for 30 s on the 6th day. An online DigBehav-Morris water maze video analysis system (Mobile Datum Software Technology) was used to record the escape latencies, the total time spent in target quadrants and the number of platform crossings of rats in this test.

### SDS–PAGE and immunoblotting

Molecular studies began on the 22nd day postsurgery. Hippocampus (bilateral) and cortex of brain were removed in each group, respectively, homogenized and extracted in cold RIPA buffer (Beyotime, China) containing protease and phosphatase inhibitor cocktail (Roche) for 30 min, then centrifuged at 13,000 g for 15 min at 4°C. Equal amounts of protein samples were separated by SDS–PAGE and transferred onto nitrocellulose filters (Merck Millipore Ltd., USA), followed by blocking with 5% skim milk. The membranes were incubated with the following primary antibodies: anti-netrin-1(ab126729, Abcam, 1:500), anti-NLRP3 (ab263899, Abcam, 1:1,000), anti-caspase-1 (A18646, Abclone, 1:500), anti-ASC (sc-514414, Santa, 1:1,000), anti-IL-1β (ab254360, Abcam, 1:1,000), anti-IL-18 (ab71495, Abcam, 1:5,000), anti-Aβ (Millipore, 1:1,000), anti-β-actin (ab8226, Abcam, 1:1,000), anti-p65 (8242, CST, 1:1,000), anti-p-p65 (3033, CST, 1:1,000), anti-Iκba (4814, CST, 1:1,000), anti-pIκba (2859, CST, 1:1,000), anti-Iba-1 (ab178847, Abcam, 1:1,000). After washing with PBST, secondary antibodies from Li-COR Biotechnology (1:10,000) were added to the samples. Immunoreactivity was detected using an Odyssey CLx imager (Licor, Bad Homburg, Germany), and pictures were analyzed using Image Studio (Licor, Bad Homburg, Germany).

### Enzyme-linked immunosorbent assay

Enzyme-linked immunosorbent assay (ELISA) kits were used for determining quantitatively cerebrospinal fluid (CSF) sAPPa (27419, IBL, Japan) and Aβ1-42 (27721, IBL, Japan) concentrations of rats, according to the protocols provided by the manufacturer. The CSF was collected from the rat by foramen magnum puncture. Briefly, 30 μL of each sample and its control were added to the 96-well microtiter plate which precoated with capture antibody and incubated overnight at 4°C. Plates were washed for 5 times and incubated for 30 min with detection antibody at room temperature, followed by added chromogen solution for 30 min. Optical densities were all measured at 450 nm.

### Statistical analysis

Statistical analyses were presented as GraphPad Prism 5.0 (GraphPad Software, La Jolla, USA). P <0.05 was considered statistically significant. Data are presented as the mean ± standard deviation. Differences between two paired groups was evaluated with *t* tests and two-way analysis of variance (ANOVA) followed by Bonferroni *post hoc* analysis were assessed among the multiple groups. GraphPad Prism 5.0 was used to construct graphs.

## Results

### NTN-1 ameliorated memory and cognitive impairment in aβ1-42-induced AD rats

**Figure 2:**
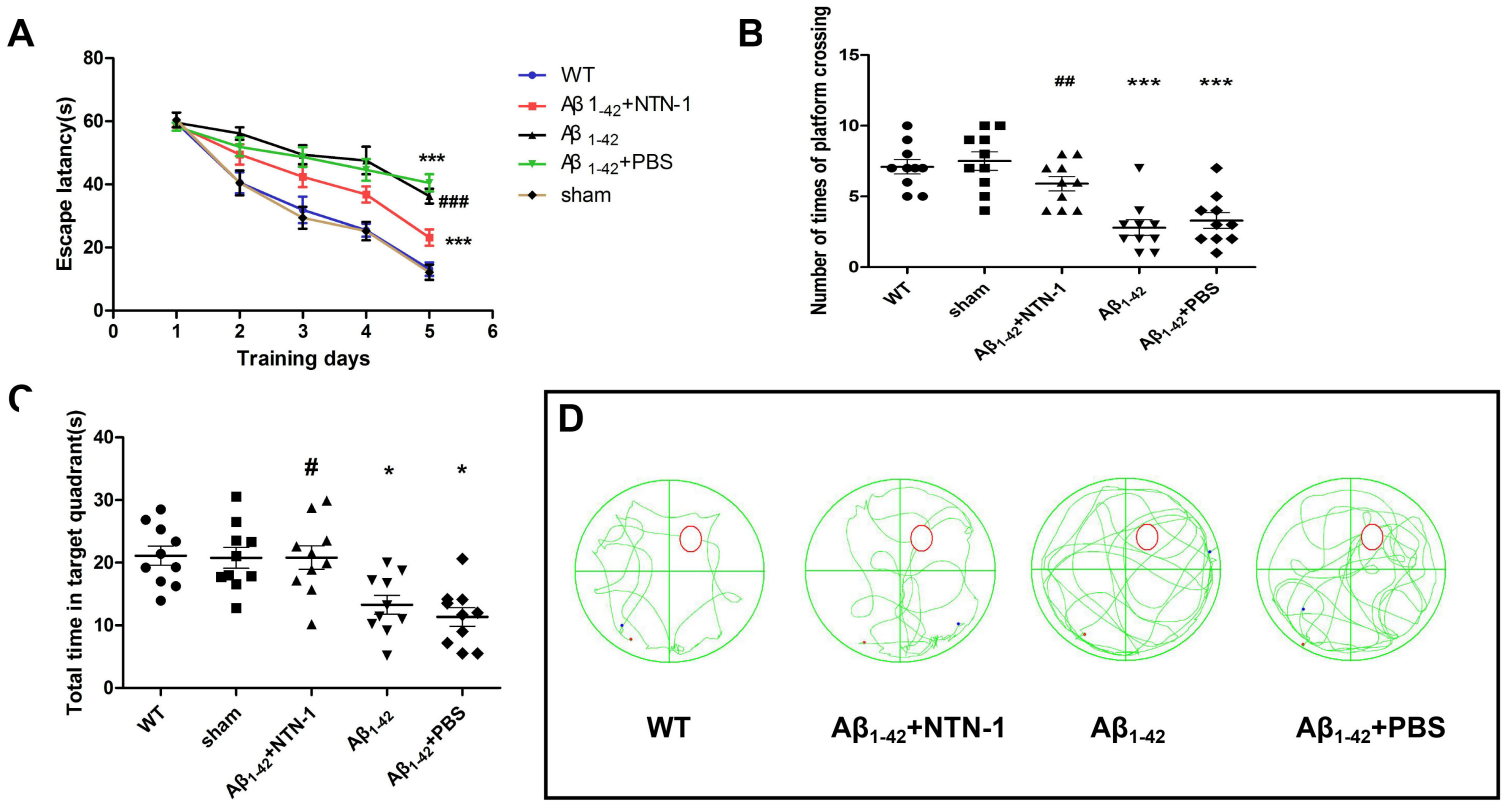
NTN-1 rescues impaired water maze learning in Aβ1-42-induced AD rats (n=10 per group). **(A)** Escape latencies in water maze phase. **(B)** The number of times of platform in the probe test. **(C)** The total time spent in the target quadrant in the test. **(D)** Representative swimming paths in the training phase. The data are presented as the mean±SD. *P < 0.05, ***P < 0.001 vs. WT, ^#^P < 0.05, ^##^P < 0.01 vs. the Aβ1-42 group. NTN-1, Netrin-1; WT, Wild-type.

The behavioral tests of rat models were measured in our experiment, as shown in Figure 2, there was no significant difference between wild-type and sham groups, however, the Aβ1-42 and Aβ1-42+PBS rats had a decreased level in the total time spending in target quadrants and the number of crossing times of platform, further more, the escape latencies were elevated for AD rats. Spatial learning and memory was significantly improved in Aβ + NTN-1 rats, along with an increased degree of total time spent in the target quadrants (*P* < 0.001) and the number of platform crossings (P <0.01) and a decreased level of escape latency (*P* < 0.05) compared with the vehicle group of AD rats (Figures 2A–C). Our results were consistent with previous studies showing that NTN-1 administration produced a clear elevation in memory and cognitive ability in Aβ1-42-induced AD rats (Zamani et al., 2019).

### The NLRP3 inflammasome and microglia were activated in aβ1-42-induced AD rat hippocampal tissues

**Figure 3:**
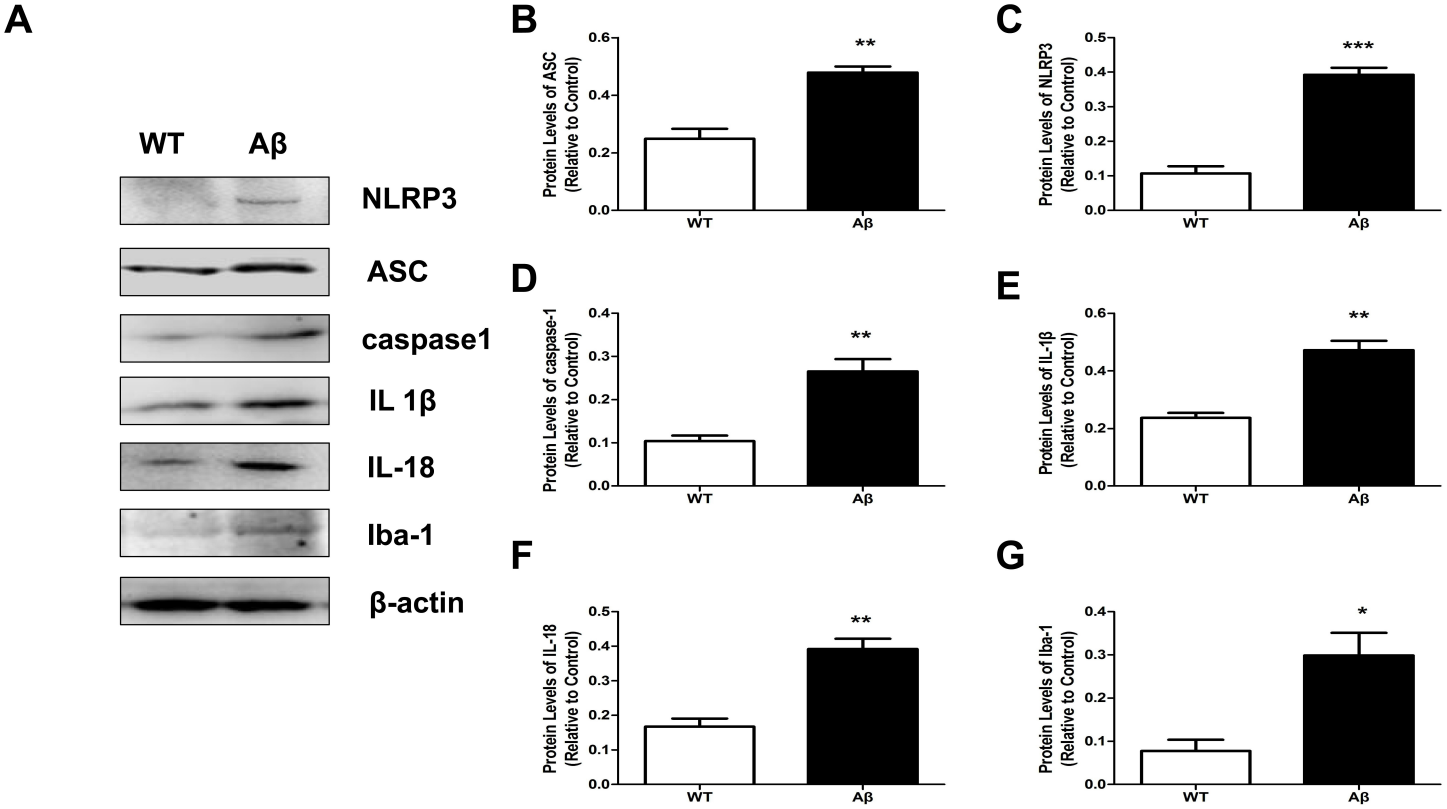
The NLRP3 inflammasome and microglia were activated in Aβ1-42-stimulated rats. The protein levels of NLRP3, ASC, caspase-1, IL-1β, and IL-18 as well as the microglial marker Iba-1 were assayed by Western blot in wild-type and Aβ1-42 rat brains (**A**, n=4 per group). The levels of proteins were quantified and normalized to the level of β-actin **(B–G)**, *P<0.05, **P<0.01 and ***P<0.001 compared with the wild-type group. Data are recorded as the mean ± SD for three independent experiments. NTN-1, Netrin-1; WT, Wild-type.

Microglia are promptly activated by vast amounts of stimuli, such as Aβ peptide. Activated microglial cells generate neuroinflammation and NLRP3 inflammasome activation and secrete a variety of proinflammatory factors, resulting in neuronal damage and psychological symptoms of AD (Jin et al., 2019). The hippocampus and cerebral cortex are densely populated areas at the junction of gray matter and white matter in the brain, with abundant neurons and microglia involved in cognition and memory. Therefore, we chose to take samples from these areas for detecting the immune activity response of microglia cells in AD. In our previous experiments utilizing an Aβ1–42-induced rat model, neurotoxicity was significantly more pronounced in the AD14d group compared to the AD7d group, indicating a temporal correlation between disease progression and the extent of neuronal damage/repair (Sun et al., 2019). To maximize observable injury and enhance NTN-1 treatment *in vivo* efficacy, tissue sampling was conducted at 22 days post-intervention. In our study, the levels of the NLRP3 inflammasome in the hippocampal tissues of wild-type and Aβ1-42 rats were tested at 22 postsurgery by Western blotting. As shown in Figure 3, compared to the WT group, the protein expression of NLRP3/ASC/caspase-1 was significantly increased in AD rat brains. Additionally, the expression of proinflammatory factors downstream (IL1β and IL18) was also upregulated in Aβ1-42 rats (*P* < 0.01). We also measured Iba-1 protein levels (a microglial marker) to detect activated microglia, which were increased in the AD group (*P* < 0.05). Similar results were also observed between sham group and AD group (data not shown). These results confirmed previous studies that microglial inflammation and the activation of NLRP3 inflammasomes result in neuropathological progression in AD, and NTN-1 might be correlated with these processes.

### NTN-1 inhibited the activation of the NLRP3 inflammasome in aβ1-42-induced AD rats

**Figure 4:**
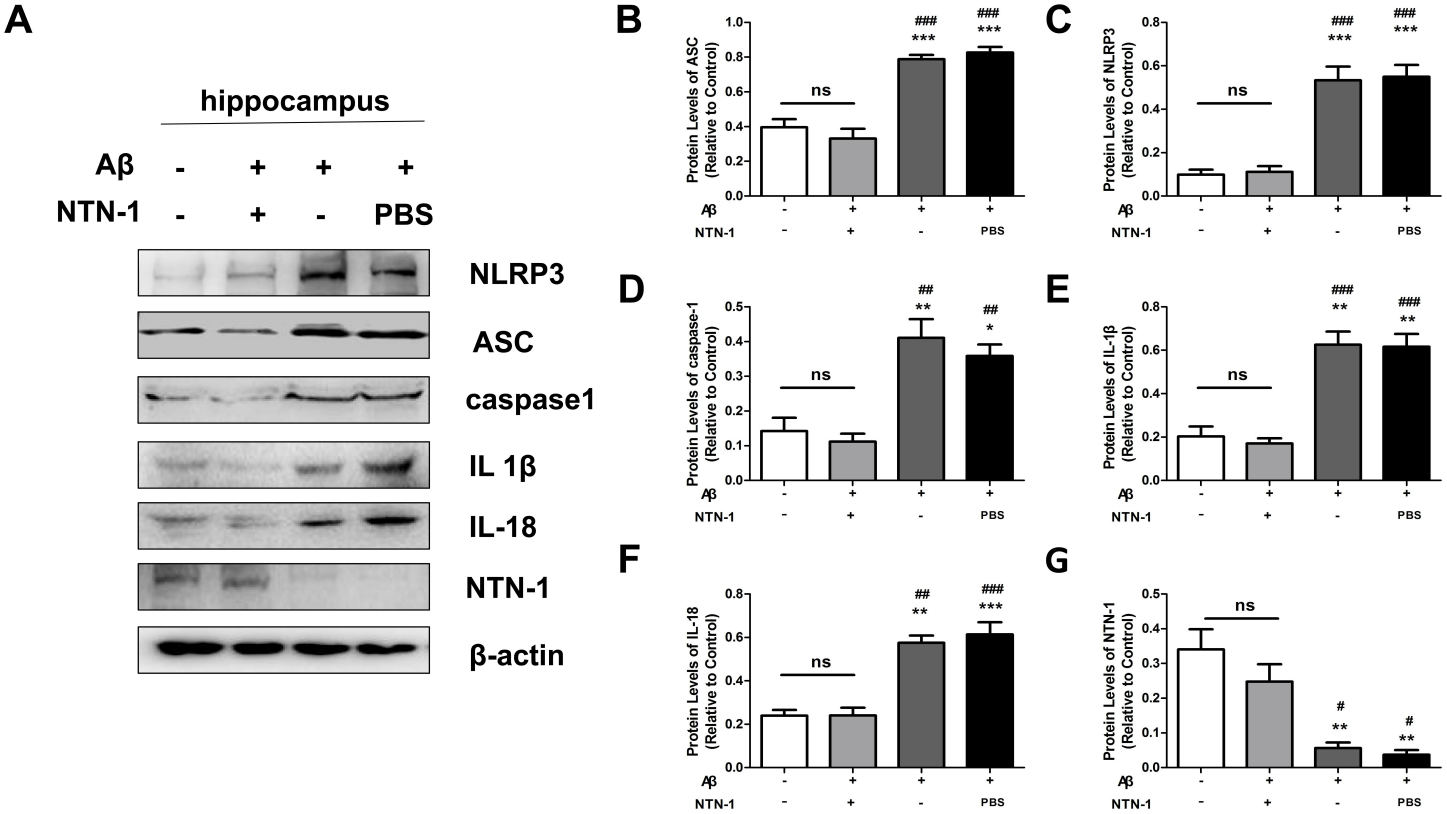
NTN-1 inhibited NLRP3 inflammasome activation in hippocampal tissues of Aβ 1-42-stimulated rats. Western blotting **(A)** and quantification **(B–G)** of the levels of NLRP3, ASC, caspase-1, IL-1β, IL-18, and NTN-1 proteins were performed in the wild-type group, Aβ1-42 group, Aβ1-42 +NTN-1 group and Aβ1-42+PBS group. Protein levels were normalized to β-actin. Data are expressed as the mean ± SD (n =4), *p < 0.05, **p < 0.01, ***p < 0.001 compared with the wild-type group, ^#^P<0.05, ^##^P<0.01, ^###^P<0.001 compared with the Aβ1-42+NTN-1 group.

**Figure 5:**
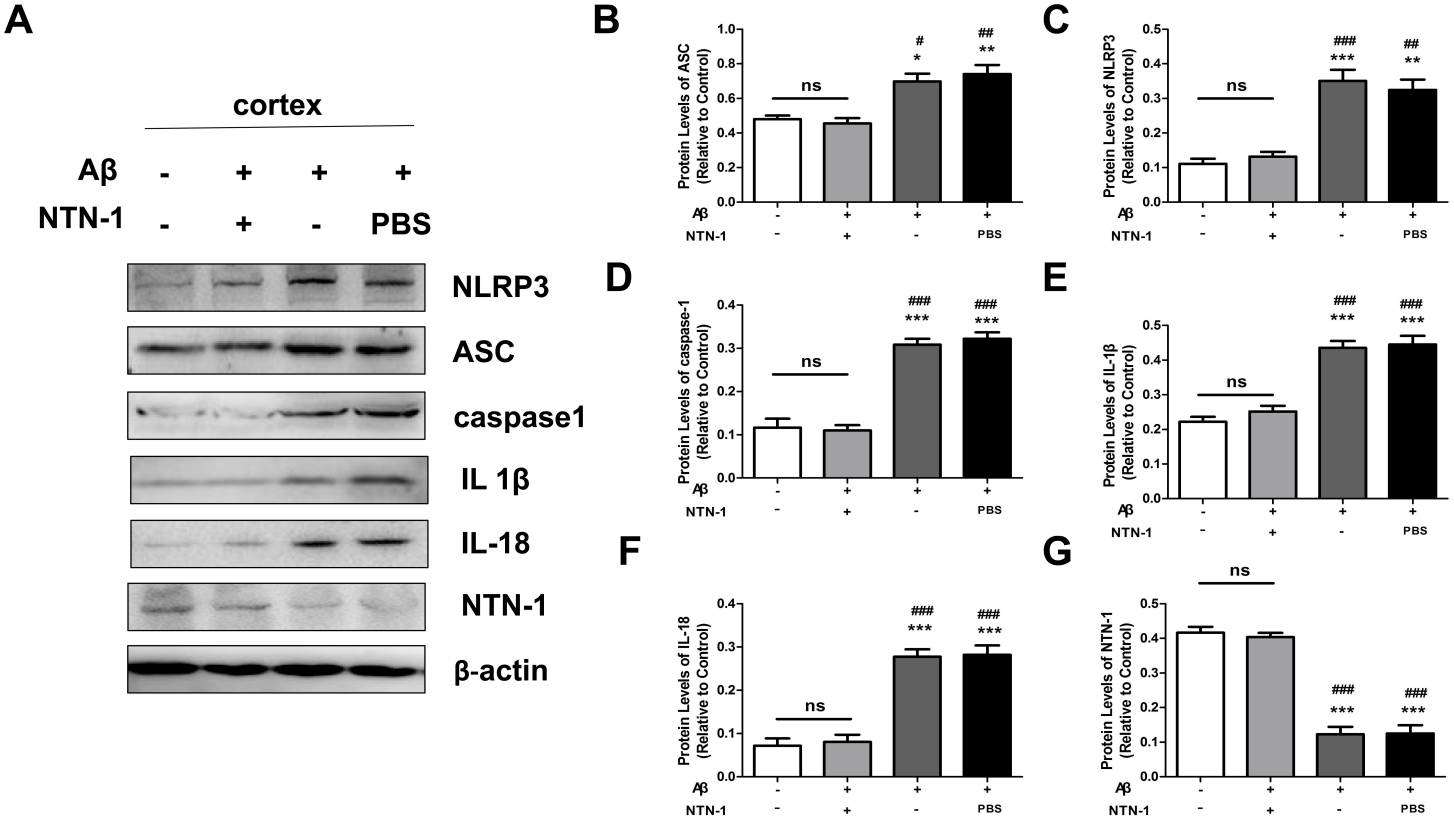
NTN-1 inhibited NLRP3 inflammasome activation in cortical tissues of Aβ1-42-stimulated rats. Western blotting **(A)** and quantification **(B–G)** of the levels of NLRP3, ASC, caspase-1, IL-1β, IL-18, and NTN-1 proteins were performed in the wild-type group, Aβ1-42 group, Aβ1-42 +NTN-1 group and Aβ1-42+PBS group. Protein levels were normalized to β-actin. Data are expressed as the mean ± SD (n =4), *p <0.05, **p < 0.01, ***p < 0.001 compared with the wild-type group, ^#^P<0.05, ^##^P<0.01, ^###^P<0.001 compared with the Aβ1-42+NTN-1 group.

Recent studies have reported that NTN-1 exhibits an anti-inflammatory effect in various diseases. To investigate the effects of NTN-1 on the Aβ1-42-induced NLRP3 inflammasome and microglial activation, age-matched adult male rats were separated into four groups: WT group, Aβ1-42 group,vehicle control group (Aβ1-42+PBS) and Aβ1-42+NTN-1 group, and Aβ1-42 or NTN-1 microinjection was measured as previously described. The hippocampal and cortical tissues were dissected to measure the protein expression of NLRP3 inflammasomes by Western blot analysis. In our results, compared with that in the WT group, the NLRP3 inflammasome was activated in the Aβ1-42 group (*P* < 0.001), and NTN-1 significantly prevented this increase in hippocampal protein expression ([*P* < 0.001, Figures 4A–G)]. Moreover, the expression of IL1β and IL18 was diminished by NTN-1 compared to the Aβ1-42 group (*P* < 0.01), and similar results were observed in the cortical tissues of each group (Figure 5). These results indicated that NTN-1 reduced the activation of microglia and NLRP3 inflammasomes in both hippocampal and cortical tissues of Aβ1-42-induced AD rats, which might exert neuroprotective effects.

### NTN-1 reduced the levels of the NF-κb signaling pathway in aβ1-42-stimulated rats

**Figure 6:**
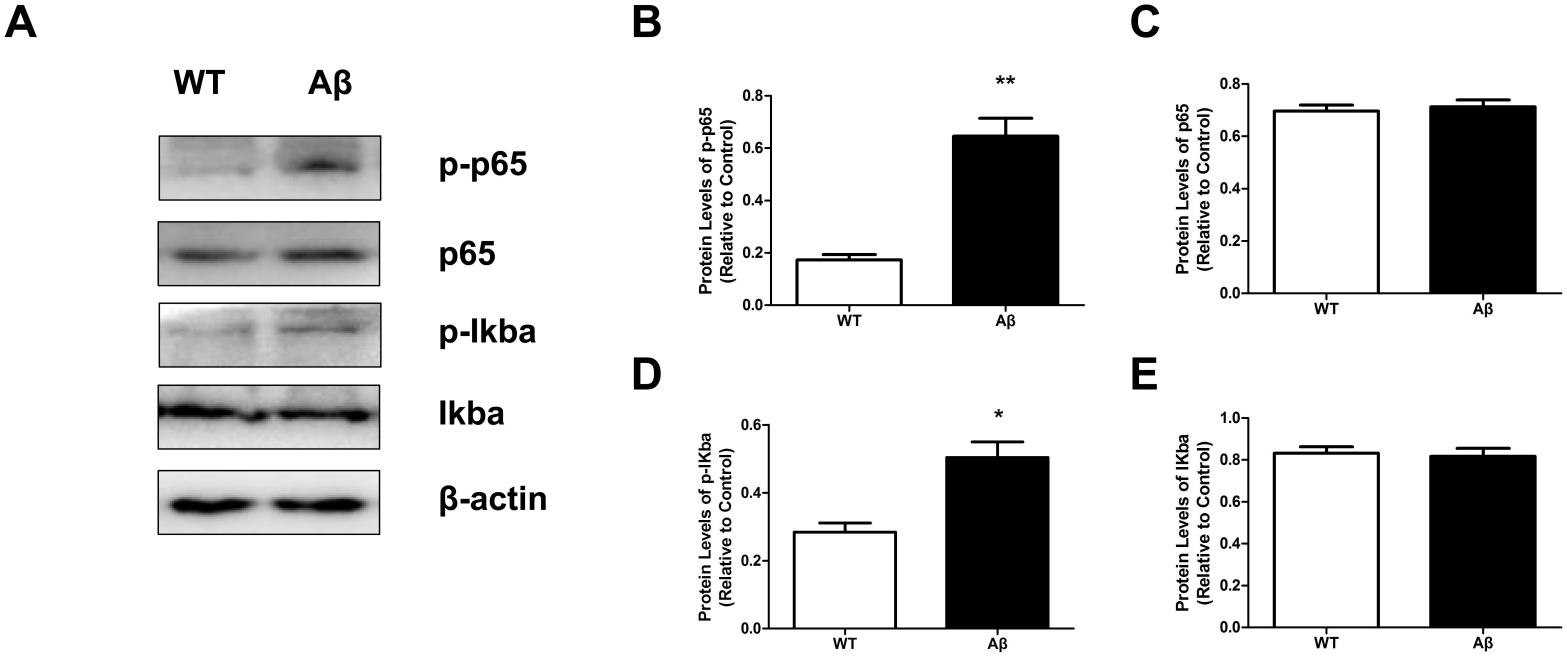
The NF-κB signaling pathway was activated in the cortex of Aβ1-42-stimulated rats. Western blot analysis of the protein levels of p-p65, p65, p-IκBα and IκBα in wild-type and Aβ1-42-stimulated rats (**A**, n=4 per group). The protein levels were quantified and normalized to the level of β-actin **(B–E)**, *p<0.05, **p<0.01 compared with the wild-type group. Data are recorded as the mean ± SD for three independent experiments. IκBα, IkappaBalpha; NF-κB, Nuclear factor-κB; Aβ, Amyloid-β; NTN-1, Netrin-1; WT, Wild-type.

**Figure 7:**
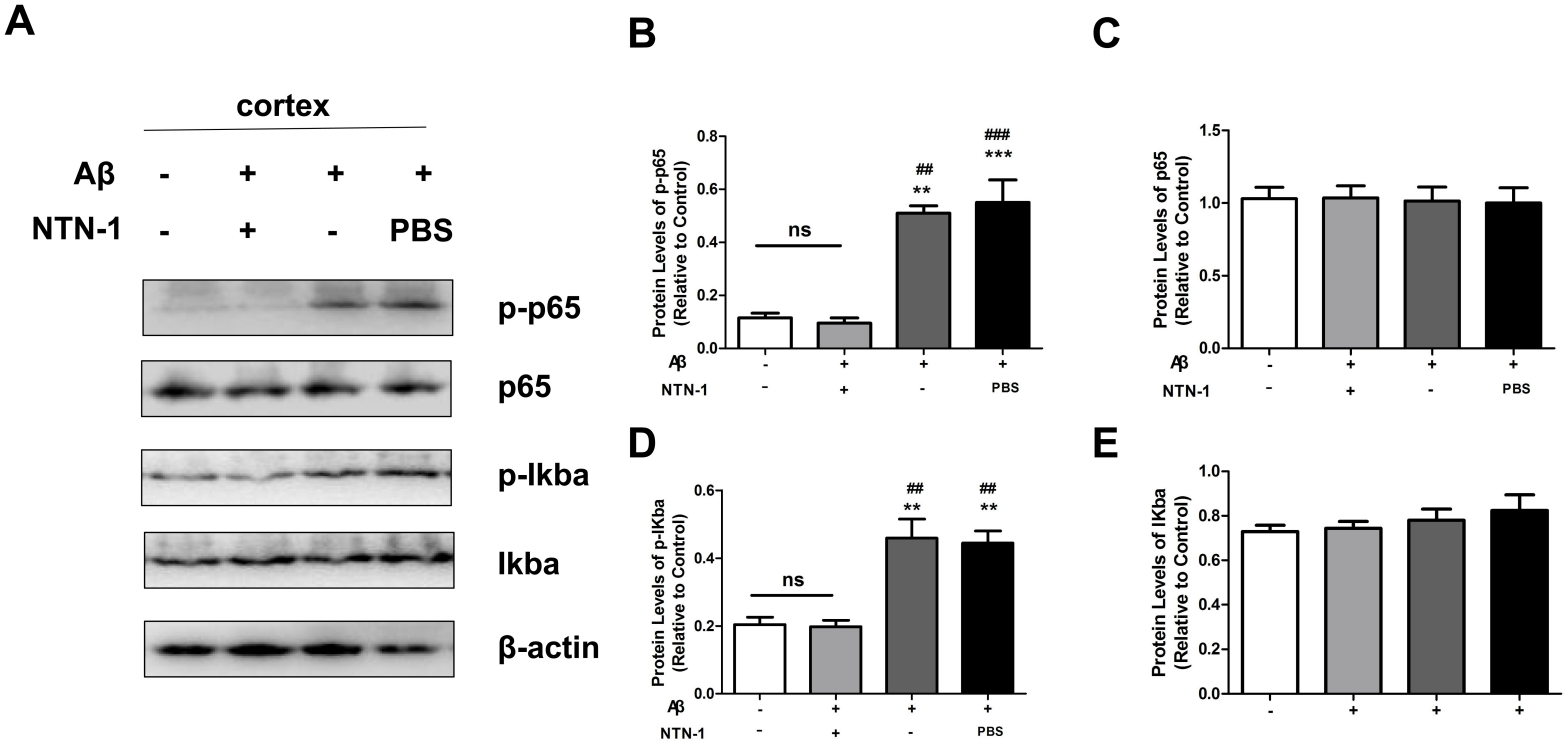
NTN-1 inhibited NF-κB activation in the cortex of Aβ1-42-stimulated rats (n=4 per group). The protein levels of p-p65, p65, p-IκBα and IκBα were measured by Western blot **(A)**, and the expression of protein was normalized to β-actin using ImageJ software **(B–E)**. **p<0.01, ***p<0.001 indicates that the expression was significantly different compared to the wild-type group, ^##^P<0.01, ^###^P<0.001 compared with the Aβ1-42+NTN-1 group. Data are recorded as the mean ± SD for three independent experiments.

The transcription factor NF-κB upregulates the expression of NLRP3, which is a critical event for NLRP3 inflammasome activation (Kelley et al., 2019). To further investigate the role of NTN-1 in NLRP3 inflammasome regulation, the protein levels of the NF-κB signaling pathway were tested in AD rats. Our results demonstrated that Aβ1-42 stimulated the phosphorylation of IκBα and NF-κB p65 expression compared with the WT group (*P* < 0.05), and NTN-1 treatment significantly inhibited Aβ-induced activation of the NF-κB pathway in rat cortex (Figures 6, 7, *P* < 0.01)). Collectively, these findings suggested that NTN-1 most likely inhibits the activation of NLRP3 inflammasomes through the NF-κB signaling pathway to reduce microglia-mediated neuroinflammation in Aβ1-42 rats.

### NTN-1 prevented an increase in aβ protein and improved the microglial microenvironment in AD rat hippocampal tissues

**Figure 8:**
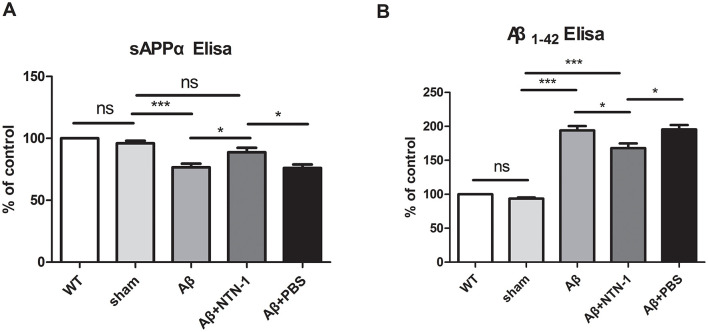
Increased levels of Aβ 1-42 and decreaed levels of sAPPα in the CSF of AD rats (*n* = 4 per group) were shown by Elisa, and NTN-1 ameliorates these effects. sAPPα **(A)** and Aβ1-42 **(B)** soluble levels were measured in CSF samples of the Aβ1-42-hippocampus microinjected rat models with treated or untreated NTN-1 using ELISA techniques. Levels were normalized to that of wild-type group (WT). Data shown as mean ± SD and differences between means were assessed using two-way ANOVA with Bonferroni multiple comparisons for three independent experiments. *p < 0.05, ***p < 0.001. CSF, Cerebrospinal fluid; sAPPα, secreted amyloid precursor protein-alpha.

Aβ1-42, dysregulated amyloidogenic pathway in amyloid precursor protein (APP) processing, is thought to be the key component of amyloid plaques and is shown to be neurotoxic in AD pathogenesis (Iwatsubo et al., 1994). It has been shown that the alternative cleavage product of APP, secreted amyloid precursor protein-alpha (sAPPα) is reduced in AD. Notably, sAPPα species has been found to exhibit many neuroprotective activities, which is important for neuron survival under pathological conditions such as AD (Bailey et al., 2011). Contrary to sAPPa, higher levels of Aβ, especially soluble Aβ 25–35 and Aβ 1–42, could lead to aggregation states with enhanced toxicity. Pathologic Aβ inhibits FL APP cleavage and decreases sAPPα production, led to the pathogenic process (Spilman et al., 2016). Soluble Aβ oligomers can stimulate chronic microglial proliferation and activation, which initiate the inflammatory cascade in AD (Jin and Yamashita, 2016). It has been shown that activated microglia can release proinflammatory cytokines, thereby reducing microglial phagocytosis, enhancing Aβ aggregation and inhibits FL APP cleavage (Pan et al., 2011). Therefore, we next evaluated the effects of NTN-1 on sAPPα and Aβ1-42 production in Aβ stimulated rats, levels of sAPPα and Aβ1-42 in rat CSF were determined by ELISA. As shown in Figure 8A we found Aβ microinjection decreased CSF sAPPα compared with WT and sham groups (*P* < 0.001), and NTN-1 significantly increased it (*P* < 0.05). In Figure 8B, ELISA analysis also showed that CSF Aβ1-42 production was elevated in AD rat (*P* < 0.001), NTN-1 treatment reversed the increase of Aβ1-42 (*P* < 0.05), which might suggest NTN-1 regulate APP processing and inhibits Aβ amplification.

**Figure 9:**
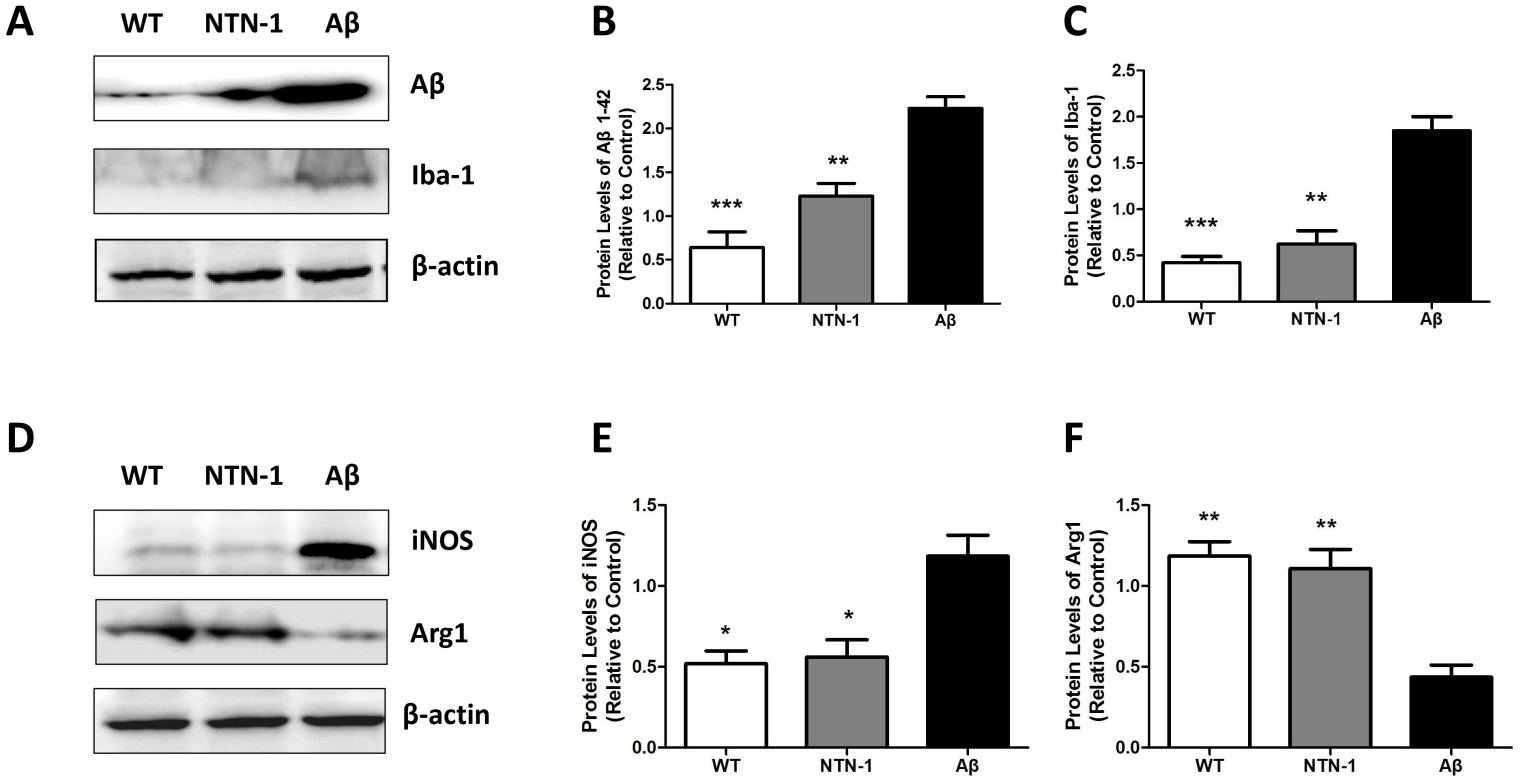
Microinjection of NTN-1 inhibited the protein expression of Aβ and altered the microglial polarization from M1 to M2. Western blot detection of the protein expression levels of Aβ and microglial marker Iba-1 **(A)**, M1 phenotype marker iNOS and M2 phenotype marker Arg1 **(D)** in the brains of the wild-type group, NTN-1 treatment group and Aβ group. Expression was normalized to that of β-actin **(B, C, E, F)**. The data are presented as the mean±SD for three independent experiments(n=4 per group). *p<0.05,**p<0.01, ***p<0.001 relative to the Aβ group.

Microglial cells play central role in degrading Aβlevel through proliferation, polarization and phagocytosis, abnormal Aβ accumulations active microglial M1 phenotypic and induce CNS inflammatory damage. Teter et al. demonstrated that Aβ stimulation induced microglial proliferation, activation, and phagocytic encapsulation of Aβ aggregates in human brain sections. Activated microglia predominantly localize to plaque cores, while peripheral regions exhibit diminished microglial density (Teter et al., 2019). However, chronic Aβ accumulation overwhelms microglial clearance capacity, leading to aberrant activation, polarization, and subsequent neuroinflammatory damage. To further verify these results, we tested the Iba-1(microglial marker) and Aβ protein levels in the hippocampus of AD rats and wild-type littermates. Microglial proliferation and Aβ accumulations were observed in the AD group, and NTN-1 treatment inhibited microglial activation and Aβ aggregation in the AD group (*P* < 0.01, Figures 9A–C). These findings imply a role of NTN-1 in affecting Aβ plaque formation in AD rats. In addition, we explore the effects of NTN-1 on the microglial M1/M2 polarization in Aβ stimulated rats, we examined the expressions of M1 and M2 phenotypic markers in the hippocampus of NTN-1 treated AD rats, the expressions of M1 phenotypic marker (iNOS) was significantly elevated in the AD group, and the expressions of M2 phenotypic marker (Arg1) was reduced compared with the WT group. Consistently, NTN-1 treatment remarkably reduced the expressions of M1 marker (*P* < 0.05)and increased the expressions of M2 marker (*P* < 0.01), compared with the AD group (Figures 9D–F). Taken together, these results of protein expression indicate that NTN-1 treatment induces microglia from M1 to M2 polarization in the hippocampus of Aβ1-42-induced AD rats, and improves the M2-mediated neuronal microenvironment.

## Discussion

Reactive microglial polarization is mechanistically linked to AD progression, driving neuroinflammatory cascades that culminate in synaptic dysfunction and cognitive decline. As resident phagocytes in the central nervous system, microglia exhibit two states during activation: the M1 state and M2 state. The M1 state is classically activated to produce proinflammatory cytokines; in contrast, the M2 state releases anti-inflammatory factors, ingesting debris and repairing CNS damage. Abnormal Aβ protein may indirectly induce CNS inflammatory damage by reacting with M1 microglia. It has been reported that without microglia present, high concentrations of abnormal Aβ protein cannot cause neuronal damage in AD (Giulian, 1999). NTN-1 acts as a new anti-inflammatory factor in the CNS to regulate immunity, angiogenesis and neuronal survival (Shabani et al., 2017). In a rat model of subarachnoid hemorrhage, administration of NTN-1 suppressed microglial activation and brain edema (Xie et al., 2018). The absence of cytotoxic effects was observed in rat cardiomyocytes treated with Netrin-1 alone, as evidenced by non-significant reductions in TUNEL positivity and LDH release, while demonstrated significantly enhanced survival of neonatal cardiomyocytes (Durrani et al., 2012). Brain slices from non-PDAPP transgenic mice were cultured with NTN-1, and there was no obvious Aβ1–40 and Aβ1–42 net productions appeared, thus it reflected that NTN-1 was safety and efficacy (Lourenço et al., 2009). another research showed that netrin-1 increased sAPPα levels in primary hippocampal mouse neurons without Aβ1-40 compared with wildtype. These studies indicate netrin-1 may exert an ameliorate sAPPα self-increasing effect in AD therapeutic (Spilman et al., 2016). Relevant studies gave evidence that NTN-1 could pass through the ventricle wall into brain tissue to generate long-term efficacy after acute injection, and support undertaking of subsequent chronic pump delivery studies (Spilman et al., 2016). This regulatory mechanism positions NTN-1 as a potential modulator of amyloidogenic pathways in neurodegenerative contexts. In our study, we observed that NTN-1 significantly improved learning and memory behavior in the water maze task, which was consistent with a previous study, and significantly inhibited microglial proliferation and activation in Aβ1-42-induced AD rats, indicating that exogenous NTN-1 treatment attenuated Aβ-induced neurological impairments in memory and learning, which may be due to M1 microglial activation in AD rats. Schwann cells were identified as the predominant source to secrete NTN-1 in the adult rat sciatic nerve. Meanwhile, NTN-1 treatment also induced Schwann cells proliferation through Unc5b receptor (Lee et al., 2007). Therefore, based on the above evidence, we speculate that exogenous injection of NTN-1 may promote myelinating oligodendrocytes proliferation and increase endogenous NTN-1 secretion in CNS. Another study suggested that NTN-1 promoted rat RSC96 Schwann cell proliferation and migration at 100 ng/mL concentration, but suppressed migration at the concentration of 500 ng/mL, this biphasic response suggested the concentration-dependent receptor signaling modulation of NTN-1. We observed that NTN-1 administration ameliorated memory and cognitive ability in Aβ1-42-induced AD rats at a concentration of 160 ng/μL, but effective concentration range of NTN-1 in the central nervous system remains further investigation (Lv et al., 2015).

Previous investigators revealed that NLRP3 inflammasome activation plays a critical role in Alzheimer's disease. Abnormal microglial activation simulated by Aβ is fundamental for activation of the NLRP3 inflammasome and subsequent proinflammatory cytokine IL-1β/IL-18 release in the brains of AD patients (Saresella et al., 2016). Furthermore, it was suggested that microglia tended to be in an M2 state and resulted in decreased deposition of Aβ in an NLRP3-/- APP/PS1 mouse model (Heneka et al., 2013). It is well documented that treatment with NTN-1 also regulates neuroinflammation and reduces blood–brain barrier disruption and disease severity (Podjaski et al., 2015). However, it remains unknown whether NTN-1 contributes to NLRP3 inflammasome activation in AD. Neurons and myelinating oligodendrocytes serve as primary cellular sources of NTN-1 synthesis and secretion within the adult CNS. In our study, there was a low level of NTN-1 in the brains of Aβ1-42-simulated AD rats. In contrast, NLRP3/ASC/caspase-1 pathway proteins were highly expressed, and the proinflammatory cytokines IL-1β and IL-18 were expressed in both hippocampal and cortical tissues, which was effectively inhibited by administration of NTN-1 by microinjection. The results indicated the impact of the NLRP3/ASC/caspase-1 pathway on AD. NTN-1 inhibited the abnormal activation of the NLRP3 inflammasome and the release of proinflammatory cytokines induced by the Aβ1-42 protein. Interestingly, the expression of NTN-1 was also diminished in the untreated Aβ1-42 group, which was consistent with our previous studies in both serum and cerebrospinal fluid compartments of AD rat and patients (Sun et al., 2019; Ju et al., 2022). Recent evidence documents significant NTN-1 downregulation in diverse pathologies, including autoimmune disorders and chronic CNS injuries (Bruikman et al., 2020). Notably, elevated Aβ concentrations promote pathological aggregation states characterized by heightened neurotoxic potential. Taken together, the decrease in NTN-1 levels may be related to the reduced in cell proliferation and secretion after nerve injury. The observed inverse correlation between NTN-1 and Aβ burden suggests that amyloid plaque progression may inhibit NTN-1 metabolic, which at least partially contributes to its accumulation in the AD brain.

To further identify the molecular mechanism related to NLRP3 inflammasome regulation in AD rats by NTN-1, we measured the relative protein expression of the NF-κB pathway by Western blot. NF-κB is a master transcription factor that generates NLRP3 and inflammatory molecules. During inflammation stimulation, activated IKK rapidly induces the phosphorylation and degradation of IκBα, followed by NF-κB p65 subunit phosphorylation, which enhances nuclear translocation and regulates the transcription of target genes, including IL-1β, IL-18, IL-6 and NLRP3 (Chang et al., 2015; Chen et al., 2016). A number of works have proposed a strong correlation between the NF-κB pathway and AD (Thawkar and Kaur, 2019). Our data were consistent with previous studies showing that the NF-κB pathway was activated by Aβ1-42 stimulation and that treatment with NTN-1 significantly inhibited Aβ1-42-induced activation of the NF-κB pathway in the brains of AD rats. It has been reported that NTN-1 signals are essential to receptors expressing in cells to survive in the extracellular environment.Unc5b, one of the receptors of NTN-1, has been widely investigated in microglia, endothelial cells, and neurons. Specific knockdown of Unc5b significantly reverses the anti-inflammation effect of NTN-1, which suppresses microglia activation (Lin et al., 2018; Xie et al., 2018). Lin et al. find that NTN-1 can bind to its receptor Unc5b and prevent NF-κB signaling in endothelial cells via an anti-inflammatory properties (Lin et al., 2018). A study has shown that connecting the NTN-1 to UNC5B via the PI3K signaling pathway exerts potent anti-apoptotic effects, significantly enhancing neuronal survival under pathological conditions (Tang et al., 2008). Interestingly, NTN-1 demonstrates cytoprotective capabilities in vascular endothelia, counteracting hyperglycemia-induced injury and angiogenic dysfunction through PI3K/AKT/eNOS pathway activation (Xing et al., 2017), this signaling cascade enhances eNOS enzymatic activity and protein expression, subsequently suppressing nuclear NF-κB translocation via eNOS/NO/NF-κB pathway (Yu et al., 2018). Based on current evidence, we hypothesize that NTN-1 lowered the Aβ1-42 induced NLRP3 inflammasome-mediated inflammatory response and microglia activation, through modulating the PI3K/eNOS/NF-kB signaling pathway in the hippocampal and cortical tissues of AD rats, which might bind to its receptor Unc5b. However, due to the multiplicity of NTN-1 receptor subtypes and binding sites, distinct functional roles may arise from different interaction sites. Multiple signaling pathways might participate in the inhibitory effects of NTN-1 on NF-kB activation, future investigation of NTN-1 and its specific receptor in regulating Aβ1-42 induced neuroinflammation injury need further investigation.

M2 microglia form a protective barrier to prevent the accumulation of Aβ plaques; however, chronic toxicity and aging conditions render M1 microglia activated in the AD brain, which causes a prolonged production of neuroinflammatory responses correlated with increased Aβ pathology in AD patients and transgenic models (Zuroff et al., 2017). It had been reported that there was an increase level of Aβ 1-42 in the CA3 hippocampal region of AD-like mice model evaluating by Immunohistochemistry Garcez et al., 2019). In addition, Zamani et al. used Congo red staining technique to qualitative assessment the effect of NTN-1 on Aβ aggregation in the AD rat hippocampal sections, the accumulated Aβ plaques are visible and significantly decreased in Aβ+NTN-1 group compared with Aβ group (Zamani et al., 2019). In our results, NTN-1 was found to decrease CSF Aβ level, increase sAPPα production, FL APP cleavage and induce microglia from M1 to M2 polarization, thereby preventing Aβ aggregates in AD rats and might be a result of alterations in neuroinflammatory impairment. Future studies will incorporate multi-timepoint assessments in transgenic AD models to evaluate dynamic changes in downstream biomarkers, thereby enhancing data clarity and robustness.

This study still has some shortcomings, our current studies focus on male rats but limit the applicability of the findings to female rats, the possible changes that may occur between different genders in the pathological process of AD are different. Early-stage Aβ pathology models (8~10-month-old adults) are selected in this study, as AD is an age-related disease, late disease models (18~22-month-old rodents) should be valued and applied in subsequent research. This experiment only applies a single drug dose (160 ng/μL) based on prior efficacy/safety data in CNS models (Zamani et al., 2019). In future studies, dose gradients should be added to establish therapeutic windows, with preliminary data showing concentration-dependent NLRP3 suppression. Single NTN-1 group without Aβ1-42 injection is lacked which help distinguish between neuroprotective and general cognitive-enhancing effects of NTN-1. Although Morris Water Maze was prioritized for its sensitivity to hippocampal-dependent spatial memory, multimodal behavioral tests would provide a more comprehensive evaluation of cognitive function. Multiple inflammatory pathways are involved in the pathological process of Alzheimer's disease, while our hypothesis-driven approach centered on NLRP3/NF-κB due to their established AD relevance, other potential inflammatory pathways, such as the JAK-STAT pathway, may be implicated in AD progression need to be further explored in the future.

## Conclusions

In conclusion, our findings suggest the beneficial effect of NTN-1 on neuroprotective and cognitive improvement in Aβ1-42-induced AD rats. In addition, the involvement of NF-κB-NLRP3 inflammasome pathway inhibition by NTN-1 might lead to a new agent for the underlying pathophysiology that is responsible for antagonizing and alleviating Alzheimer's disease.

## Data Availability

The original contributions presented in the study are included in the article/supplementary material, further inquiries can be directed to the corresponding author.

## References

[B1] Ai J. Sun L. H. Che H. Zhang R. Zhang T. Z. Wu W. C.. (2013). MicroRNA-195 protects against dementia induced by chronic brain hypoperfusion via its anti-amyloidogenic effect in rats. J. Neurosci. 33, 3989–4001. 10.1523/JNEUROSCI.1997-12.2013 23447608 PMC6619292

[B2] Bailey J. A. Ray B. Greig N. H. Lahiri D. K. (2011). Rivastigmine lowers Aβ and increases sAPPα levels, which parallel elevated synaptic markers and metabolic activity in degenerating primary rat neurons. PLoS One 6:e21954. 10.1371/journal.pone.0021954 21799757 PMC3142110

[B3] Bauernfeind F. G. Horvath G. Stutz A. Alnemri E. S. MacDonald K. Speert D.. (2009). Cutting edge: NF-kappaB activating pattern recognition and cytokine receptors license NLRP3 inflammasome activation by regulating NLRP3 expression. J. Immunol. 183, 787–791. 10.4049/jimmunol.0901363 19570822 PMC2824855

[B4] Bruikman C. S. Vreeken D. Hoogeveen R. M. Bom M. J. Danad I. Pinto-Sietsma S. J.. (2020). Netrin-1 and the grade of atherosclerosis are inversely correlated in humans. Arterioscler. Thromb. Vasc. Biol. 40, 462–472. 10.1161/ATVBAHA.119.313624 31801376

[B5] Chang X. Luo F. Jiang W. Zhu L. Gao J. He H.. (2015). Protective activity of salidroside against ethanol-induced gastric ulcer via the MAPK/NF-κB pathway *in vivo* and *in vitro*. Int. Immunopharmacol. 28, 604–615. 10.1016/j.intimp.2015.07.031 26241782

[B6] Chen T. Wang R. Jiang W. Wang H. Xu A. Lu G.. (2016). Protective effect of astragaloside IV against paraquat-induced lung injury in mice by suppressing rho signaling. Inflammation 39, 483–492. 10.1007/s10753-015-0272-4 26452991

[B7] Dun X. P. Parkinson D. B. (2017). Role of netrin-1 signaling in nerve regeneration. Int. J. Mole. Sci. 18:491. 10.3390/ijms18030491 28245592 PMC5372507

[B8] Durrani S. Haider K. H. Ahmed R. P. Jiang S. Ashraf M. (2012). Cytoprotective and proangiogenic activity of ex-vivo netrin-1 transgene overexpression protects the heart against ischemia/reperfusion injury. Stem Cells Dev. 21, 1769–1778. 10.1089/scd.2011.0475 21936706 PMC3376469

[B9] El-Gamal R. Mokhtar N. Ali-El-Dein B. Baiomy A. A. Aboazma S. M. (2020). Netrin-1: a new promising diagnostic marker for muscle invasion in bladder cancer. Urol. Oncol. 38, 640 e641–640 e612. 10.1016/j.urolonc.2020.02.006 32156466

[B10] Garcez M. L. Mina F. Bellettini-Santos T. da Luz A. P. Schiavo G. L. Macieski J. M. C.. (2019). The involvement of NLRP3 on the effects of minocycline in an AD-like pathology induced by β-amyloid oligomers administered to mice. Mol. Neurobiol. 56, 2606–2617. 10.1007/s12035-018-1211-9 30051350

[B11] Giulian D. (1999). Microglia and the immune pathology of Alzheimer disease. Am. J. Hum. Genet. 65, 13–18. 10.1086/302477 10364511 PMC1378069

[B12] Halle A. Hornung V. Petzold G. C. Stewart C. R. Monks B. G. Reinheckel T.. (2008). The NALP3 inflammasome is involved in the innate immune response to amyloid-beta. Nat. Immunol. 9, 857–865. 10.1038/ni.1636 18604209 PMC3101478

[B13] Hansen D. V. Hanson J. E. Sheng M. (2018). Microglia in Alzheimer's disease. J. Cell Biol. 217, 459–472. 10.1083/jcb.201709069 29196460 PMC5800817

[B14] He X. Liu Y. Lin X. Yuan F. Long D. Zhang Z.. (2018). Netrin-1 attenuates brain injury after middle cerebral artery occlusion via downregulation of astrocyte activation in mice. J. Neuroinflamm. 15:268. 10.1186/s12974-018-1291-5 30227858 PMC6145326

[B15] Heneka M. T. Kummer M. P. Stutz A. Delekate A. Schwartz S. Vieira-Saecker A.. (2013). NLRP3 is activated in Alzheimer's disease and contributes to pathology in APP/PS1 mice. Nature 493, 674–678. 10.1038/nature11729 23254930 PMC3812809

[B16] Iwatsubo T. Odaka A. Suzuki N. Mizusawa H. Nukina N. Ihara Y.. (1994). Visualization of a beta 42(43) and a beta 40 in senile plaques with end-specific a beta monoclonals: evidence that an initially deposited species is a beta 42(43). Neuron 13, 45–53. 10.1016/0896-6273(94)90458-8 8043280

[B17] Jiang Q. Yi M. Guo Q. Wang C. Wang H. Meng S.. (2015). Protective effects of polydatin on lipopolysaccharide-induced acute lung injury through TLR4-MyD88-NF-κB pathway. Int. Immunopharmacol. 29, 370–376. 10.1016/j.intimp.2015.10.027 26507165

[B18] Jin X. Liu M-. Y. Zhang D-. F. Zhong X. Du K. Qian P.. (2019). Baicalin mitigates cognitive impairment and protects neurons from microglia-mediated neuroinflammation via suppressing NLRP3 inflammasomes and TLR4/NF-κB signaling pathway. CNS Neurosci. Ther. 25, 575–590. 10.1111/cns.13086 30676698 PMC6488900

[B19] Jin X. Yamashita T. (2016). Microglia in central nervous system repair after injury. J. Biochem. 159, 491–496. 10.1093/jb/mvw009 26861995

[B20] Ju T. Sun L. Fan Y. Wang T. Liu Y. Liu D.. (2022). Decreased netrin-1 in mild cognitive impairment and Alzheimer's disease patients. Front. Aging Neurosci. 13:762649. 10.3389/fnagi.2021.762649 35250531 PMC8888826

[B21] Kelley N. Jeltema D. Duan Y. He Y. (2019). The NLRP3 inflammasome: an overview of mechanisms of activation and regulation. Int. J. Mole. Sci. 20:3328. 10.3390/ijms20133328 31284572 PMC6651423

[B22] Lane C. A. Hardy J. Schott J. M. (2018). Alzheimer's disease. Eur. J. Neurol. 25, 59–70. 10.1111/ene.13439 28872215

[B23] Lee H. K. Seo I. A. Seo E. Seo S. Y. Lee H. J. Park H. T.. (2007). Netrin-1 induces proliferation of Schwann cells through Unc5b receptor. Biochem. Biophys. Res. Commun. 362, 1057–1062. 10.1016/j.bbrc.2007.08.143 17825258

[B24] Li C. Zhao B. Lin C. Gong Z. An X. (2019). TREM2 inhibits inflammatory responses in mouse microglia by suppressing the PI3K/NF-κB signaling. Cell Biol. Int. 43, 360–372. 10.1002/cbin.10975 29663649 PMC7379930

[B25] Lin Z. Jin J. Bai W. Li J. Shan X. (2018). Netrin-1 prevents the attachment of monocytes to endothelial cells via an anti-inflammatory effect. Mol. Immunol. 103, 166–172. 10.1016/j.molimm.2018.08.021 30290313

[B26] Lou X. H. Cai Y. Y. Yang X. Q. Zheng H. J. Yu Y. J. Wang C. H.. (2020). Serum netrin-1 concentrations are associated with clinical outcome in acute intracerebral hemorrhage. Clin. Chim. Acta 508, 154–160. 10.1016/j.cca.2020.05.032 32417215

[B27] Lourenço F. C. Galvan V. Fombonne J. Corset V. Llambi F. Müller U.. (2009). Netrin-1 interacts with amyloid precursor protein and regulates amyloid-beta production. Cell Death Differ. 16, 655–663. 10.1038/cdd.2008.191 19148186 PMC2757418

[B28] Lv J. Sun X. Ma J. Ma X. Zhang Y. Li F.. (2015). Netrin-1 induces the migration of Schwann cells via p38 MAPK and PI3K-Akt signaling pathway mediated by the UNC5B receptor. Biochem. Biophys. Res. Commun. 464, 263–268. 10.1016/j.bbrc.2015.06.140 26116534

[B29] Moon C. Kim H. Ahn M. Jin J. K. Wang H. Matsumoto Y.. (2006). Enhanced expression of netrin-1 protein in the sciatic nerves of Lewis rats with experimental autoimmune neuritis: possible role of the netrin-1/DCC binding pathway in an autoimmune PNS disorder. J. Neuroimmunol. 172, 66–72. 10.1016/j.jneuroim.2005.11.002 16337279

[B30] Mulero P. Córdova C. Hernández M. Martín R. Gutiérrez B. Muñoz J. C.. (2017). Netrin-1 and multiple sclerosis: a new biomarker for neuroinflammation? Eur. J. Neurol. 24, 1108–1115. 10.1111/ene.13340 28677863

[B31] Nanjundaiah S. Chidambaram H. Chandrashekar M. Chinnathambi S. (2021). Role of microglia in regulating cholesterol and tau pathology in Alzheimer's disease. Cell. Mol. Neurobiol. 41, 651–668. 10.1007/s10571-020-00883-6 32468440 PMC11448617

[B32] Pan X. D. Zhu Y. G. Lin N. Zhang J. Ye Q. Y. Huang H. P.. (2011). Microglial phagocytosis induced by fibrillar β-amyloid is attenuated by oligomeric β-amyloid: implications for Alzheimer's disease. Mol. Neurodegener. 6:45. 10.1186/1750-1326-6-45 21718498 PMC3149591

[B33] Podjaski C. Alvarez J. I. Bourbonniere L. Larouche S. Terouz S. Bin J. M.. (2015). Netrin 1 regulates blood-brain barrier function and neuroinflammation. Brain 138, 1598–1612. 10.1093/brain/awv092 25903786 PMC4614143

[B34] Saresella M. La Rosa F. Piancone F. Zoppis M. Marventano I. Calabrese E.. (2016). The NLRP3 and NLRP1 inflammasomes are activated in Alzheimer's disease. Mol. Neurodegener. 11:23. 10.1186/s13024-016-0088-1 26939933 PMC4778358

[B35] Shabani M. Haghani M. Tazangi P. E. Bayat M. Shid Moosavi S. M. Ranjbar H.. (2017). Netrin-1 improves the amyloid-β-mediated suppression of memory and synaptic plasticity. Brain Res. Bull. 131, 107–116. 10.1016/j.brainresbull.2017.03.015 28389207

[B36] Spel L. Martinon F. (2020). Inflammasomes contributing to inflammation in arthritis. Immunol. Rev. 294, 48–62. 10.1111/imr.12839 31944344

[B37] Spilman P. R. Corset V. Gorostiza O. Poksay K. S. Galvan V. Zhang J.. (2016). Netrin-1 interrupts amyloid-β amplification, increases sAβPPα *in vitro* and *in vivo*, and improves cognition in a mouse model of Alzheimer's disease. J. Alzheimers Dis. 52, 223–242. 10.3233/JAD-151046 27060954

[B38] Sun L. Ju T. Wang T. Zhang L. Ding F. Zhang Y.. (2019). Decreased netrin-1 and correlated Th17/Tregs balance disorder in Aβ(1-42) INDUCED Alzheimer's disease model rats. Front. Aging Neurosci. 11, 124–124. 10.3389/fnagi.2019.00124 31191297 PMC6548067

[B39] Tadagavadi R. K. Wang W. Ramesh G. (2010). Netrin-1 regulates Th1/Th2/Th17 cytokine production and inflammation through UNC5B receptor and protects kidney against ischemia-reperfusion injury. J. Immunol. 185, 3750–3758. 10.4049/jimmunol.1000435 20693423

[B40] Tang X. Jang S. W. Okada M. Chan C. B. Feng Y. Liu Y.. (2008). Netrin-1 mediates neuronal survival through PIKE-L interaction with the dependence receptor UNC5B. Nat. Cell Biol. 10, 698–706. 10.1038/ncb1732 18469807 PMC2839190

[B41] Teter B. Morihara T. Lim G. P. Chu T. Jones M. R. Zuo X.. (2019). Curcumin restores innate immune Alzheimer's disease risk gene expression to ameliorate Alzheimer pathogenesis. Neurobiol. Dis. 127, 432–448. 10.1016/j.nbd.2019.02.015 30951849 PMC8092921

[B42] Thawkar B. S. Kaur G. (2019). Inhibitors of NF-κB and P2X7/NLRP3/caspase 1 pathway in microglia: novel therapeutic opportunities in neuroinflammation induced early-stage Alzheimer's disease. J. Neuroimmunol. 326, 62–74. 10.1016/j.jneuroim.2018.11.010 30502599

[B43] Tschopp J. Schroder K. (2010). NLRP3 inflammasome activation: the convergence of multiple signalling pathways on ROS production? Nat. Rev. Immunol. 10, 210–215. 10.1038/nri2725 20168318

[B44] Webers A. Heneka M. T. Gleeson P. A. (2020). The role of innate immune responses and neuroinflammation in amyloid accumulation and progression of Alzheimer's disease. Immunol. Cell Biol. 98, 28–41. 10.1111/imcb.12301 31654430

[B45] Xie Z. Huang L. Enkhjargal B. Reis C. Wan W. Tang J.. (2018). Recombinant netrin-1 binding UNC5B receptor attenuates neuroinflammation and brain injury via PPARγ/NFκB signaling pathway after subarachnoid hemorrhage in rats. Brain Behav. Immun. 69, 190–202. 10.1016/j.bbi.2017.11.012 29162556 PMC5894358

[B46] Xing Y. Lai J. Liu X. Zhang N. Ming J. Liu H.. (2017). Netrin-1 restores cell injury and impaired angiogenesis in vascular endothelial cells upon high glucose by PI3K/AKT-eNOS. J. Mol. Endocrinol. 58, 167–177. 10.1530/JME-16-0239 28250059

[B47] Yu L. Yin M. Yang X. Lu M. Tang F. Wang H.. (2018). Calpain inhibitor I attenuates atherosclerosis and inflammation in atherosclerotic rats through eNOS/NO/NF-κB pathway. Can. J. Physiol. Pharmacol. 96, 60–67. 10.1139/cjpp-2016-0652 28758430

[B48] Zamani E. Parviz M. Roghani M. Hosseini M. Mohseni-Moghaddam P. Nikbakhtzadeh M.. (2020). Netrin-1 protects the SH-SY5Y cells against amyloid beta neurotoxicity through NF-κB/Nrf2 dependent mechanism. Mol. Biol. Rep. 47, 9271–9277. 10.1007/s11033-020-05996-1 33206363

[B49] Zamani E. Parviz M. Roghani M. Mohseni-Moghaddam P. (2019). Key mechanisms underlying netrin-1 prevention of impaired spatial and object memory in Aβ(1-42) CA1-injected rats. Clin. Exp. Pharmacol. Physiol. 46, 86–93. 10.1111/1440-1681.13020 30066400

[B50] Zhang J. Ke K-. F. Liu Z. Qiu Y-. H. Peng Y-. P. (2013). Th17 cell-mediated neuroinflammation is involved in neurodegeneration of aβ1-42-induced Alzheimer's disease model rats. PLoS One 8:e75786. 10.1371/journal.pone.0075786 24124514 PMC3790825

[B51] Zuroff L. Daley D. Black K. L. Koronyo-Hamaoui M. (2017). Clearance of cerebral Aβ in Alzheimer's disease: reassessing the role of microglia and monocytes. Cell. Mol. Life Sci. 74, 2167–2201. 10.1007/s00018-017-2463-7 28197669 PMC5425508

